# Vitamin C Restricts the Emergence of Acquired Resistance to EGFR-Targeted Therapies in Colorectal Cancer

**DOI:** 10.3390/cancers12030685

**Published:** 2020-03-14

**Authors:** Annalisa Lorenzato, Alessandro Magrì, Vittoria Matafora, Valentina Audrito, Pamela Arcella, Luca Lazzari, Monica Montone, Simona Lamba, Silvia Deaglio, Salvatore Siena, Andrea Bertotti, Livio Trusolino, Angela Bachi, Federica Di Nicolantonio, Alberto Bardelli, Sabrina Arena

**Affiliations:** 1 Candiolo Cancer Institute, FPO-IRCCS, Candiolo 10060 (TO), Italy; annalisa.lorenzato@unito.it (A.L.); alessandro.magri@unito.it (A.M.); pamela.arcella@unito.it (P.A.); monica.montone@ircc.it (M.M.); simona.lamba@ircc.it (S.L.); andrea.bertotti@unito.it (A.B.); livio.trusolino@unito.it (L.T.); federica.dinicolantonio@unito.it (F.D.N.); alberto.bardelli@unito.it (A.B.); 2 Department of Oncology, University of Turin, Candiolo 10060 (TO), Italy; 3 IFOM-FIRC Institute of Molecular Oncology, Via Adamello 16, Milan 20139, Italy; vittoria.matafora@ifom.eu (V.M.); luca.lazzari@ifom.eu (L.L.); angela.bachi@ifom.eu (A.B.); 4 Department of Medical Sciences, University of Turin, Turin 10126, Italy; valentina.audrito@unito.it (V.A.); silvia.deaglio@unito.it (S.D.); 5 Niguarda Cancer Center, Grande Ospedale Metropolitano Niguarda, Milan 20162, Italy; salvatore.siena@unimi.it; 6 Department of Oncology and Hemato-Oncology, Università degli Studi di Milano, Milan 20133, Italy

**Keywords:** colorectal cancer, cetuximab, drug resistance, Vitamin C, glucose metabolism, oxidative stress, ROS, ferroptosis

## Abstract

The long-term efficacy of the Epidermal Growth Factor Receptor (EGFR)-targeted antibody cetuximab in advanced colorectal cancer (CRC) patients is limited by the emergence of drug-resistant (persister) cells. Recent studies in other cancer types have shown that cells surviving initial treatment with targeted agents are often vulnerable to alterations in cell metabolism including oxidative stress. Vitamin C (VitC) is an antioxidant agent which can paradoxically trigger oxidative stress at pharmacological dose. Here we tested the hypothesis that VitC in combination with cetuximab could restrain the emergence of secondary resistance to EGFR blockade in CRC *RAS/BRAF* wild-type models. We found that addition of VitC to cetuximab impairs the emergence of drug persisters, limits the growth of CRC organoids, and significantly delays acquired resistance in CRC patient-derived xenografts. Mechanistically, proteomic and metabolic flux analysis shows that cetuximab blunts carbohydrate metabolism by blocking glucose uptake and glycolysis, beyond promoting slow but progressive ROS production. In parallel, VitC disrupts iron homeostasis and further increases ROS levels ultimately leading to ferroptosis. Combination of VitC and cetuximab orchestrates a synthetic lethal metabolic cell death program triggered by ATP depletion and oxidative stress, which effectively limits the emergence of acquired resistance to anti-EGFR antibodies. Considering that high-dose VitC is known to be safe in cancer patients, our findings might have clinical impact on CRC patients treated with anti-EGFR therapies.

## 1. Introduction

In a subset of advanced colorectal cancers (CRCs), treatment of *RAS* wild-type (wt) tumors with the anti-EGFR antibodies cetuximab or panitumumab leads to the killing of drug-sensitive cells and tumor volume reduction [1]. Unfortunately, the effect is transitory and the emergence of drug-resistant cells almost invariably leads to clinical relapses [2,3]. Several strategies have been considered to overcome secondary resistance to EGFR blockade, including vertical targeting the EGFR-RAS-MEK axis with multiple drugs. For example, our laboratory and others found that combinatorial treatment with EGFR antibodies and MEK inhibitors effectively restricts the emergence of drug resistance in CRC preclinical models [4]. While trials with these agents are still ongoing in RAS wt patients (NCT03087071, NCT02399943), previous phase I clinical studies in *RAS/BRAF* mutant patients indicate that combination of EGFR-targeted antibodies and MEK inhibitors could be limited by treatment-related toxicity [5,6]. This is likely due to the fact that several organs (such as the skin and the gut) rely on the EGFR-RAS-MEK signaling pathways in adult life and this limits the therapeutic index of inhibiting multiple nodes of EGFR signaling [7].

In the present study we considered how to restrict the emergence of secondary resistance to cetuximab while limiting side effects. We reasoned that to prolong the response to EGFR blockade, concomitant or sequential therapies should ideally target those cells that survive the initial anti-EGFR treatment, which are often referred to as ‘persister’. Persister cells are thought to represent the reservoir from which permanently resistant clones eventually emerge. Persisters are characterized by a drug-tolerant state and rely on not completely characterized genetic, epigenetic, or metabolic rewiring for their survival [8,9]. Recent evidence indicates that persister cells show increased vulnerability to oxidative stress [10,11,12]. We reasoned that Vitamin C (VitC), a water-soluble organic compound that acts as a pro-oxidant molecule when administered at pharmacological concentrations (0.1–100 mM), might be valuable in targeting persisters [13,14]. Furthermore, recent data indicate that VitC kills CRC cells carrying *RAS*-oncogenic mutations [15,16,17]. Relevant for our working hypothesis, mutations in *RAS* itself or its effectors are known mechanisms of acquired resistance to anti-EGFR antibody therapies in colorectal cancers [3]. Prompted by these data, we reasoned that the pharmacological properties of VitC, coupled with the transiently vulnerable state of cetuximab persister cells, could be exploited to target clones surviving anti-EGFR treatment, extend the clinical efficacy of cetuximab, and possibly restrict the emergence of acquired resistance to EGFR blockade. This hypothesis has never been previously tested and is highly attractive considering that high-dose VitC is known to be safe and well tolerated by cancer patients [18,19,20,21,22].

## 2. Results

### 2.1. Cetuximab-Persister Cells are Vulnerable to Vitamin C-Mediated Oxidative Stress

We selected a number of in vitro and in vivo preclinical CRC *RAS/BRAF* wt models to mimic the clinical setting in which EGFR blockade is used and tested our hypotheses by treating them with VitC and cetuximab alone or in combination.

**Figure 1:**
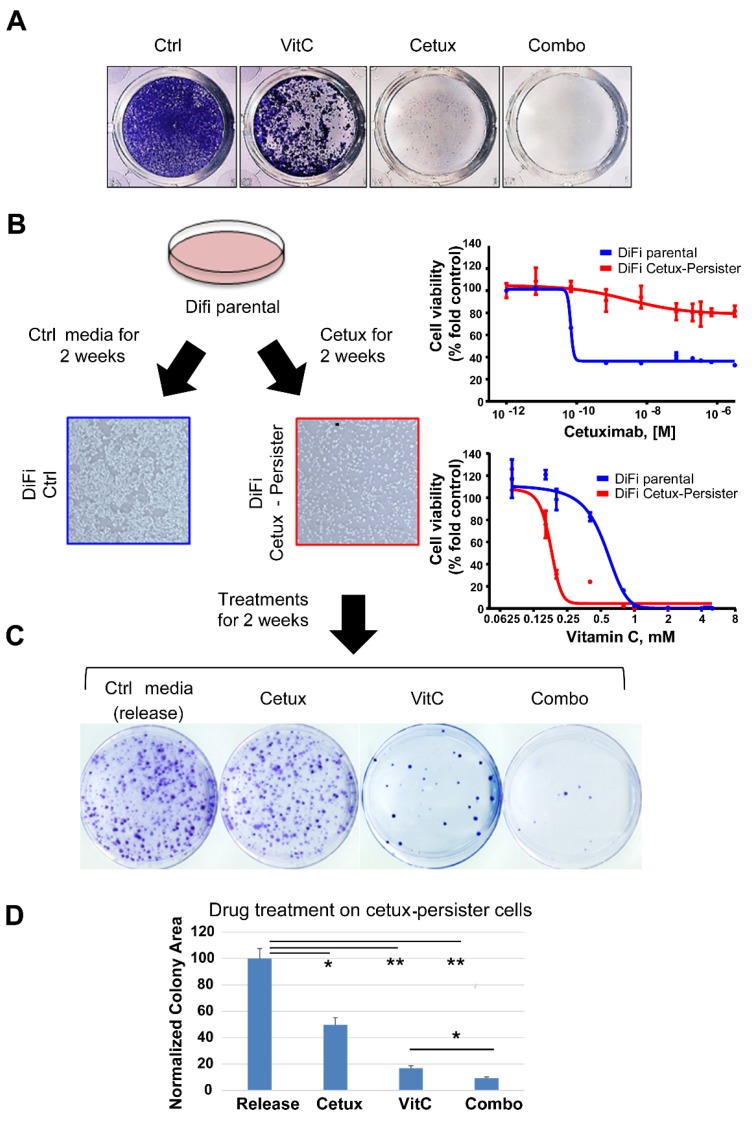
Effects of Vitamin C (VitC) treatment on cetuximab-persister colorectal cancer (CRC) cells. (**A**) DiFi cells were seeded (25,000 cells/well) in 24-well plates for a long-term proliferation assay under treatment with VitC (1 mM), cetuximab (50 μg/mL), or their combination. When cells seeded in the control wells reached confluence, all wells were fixed with paraformaldehyde and stained with crystal violet. Representative images from one of three independent experiments are shown. See also Figure S1 for results in CCK81 CRC cells. (**B**) Generation of DiFi cetuximab-persister cells (red square) by treating for 2 weeks with cetuximab (100 μg/mL), 5 million DiFi cells were seeded in 10 cm plastic plates (left part) and tested for sensitivity to cetuximab and VitC after 2 weeks of cetuximab treatment (right part). DiFi parental cells treated with control media were used as a control (blue square). (**C**) Cetuximab-persister cells were further challenged with the indicated treatment (Cetux: 100 μg/mL; VitC: 2 mM) for two more weeks and then fixed and stained with crystal violet for colony assessment. Representative images are shown from one of three independent experiments. (**D**) Colony area was calculated by the ImageJ software and numbers were normalized on control media (release) treatment. Error bars represent SD. Ctrl, control; Cetux, cetuximab; VitC, Vitamin C; Combo, combination of VitC and cetuximab; release, control media. Statistical significance: *p < 0.05; **p < 0.01; (two-tailed unpaired Student’s *t* test).

We initially considered cetuximab-sensitive 2D CRC cells (DiFi and CCK81). Treatment with VitC or cetuximab as single agents impaired DiFi cell growth at different levels, but in both instances a population of surviving cells was consistently detected (Figure 1A and Figure S1). On the contrary, combinatorial treatment abrogated the persistence of resistant cells (Figure 1A and Figure S1). We then performed a clonogenic assay where we first generated, by chronically treating cells for 2 weeks, a pool of cetuximab-persister cells (Figure 1B) that we next challenged with either VitC, cetuximab, or their combination (Figure 1C). Cetuximab-tolerant cells were more sensitive to VitC-induced oxidative stress compared to their parental counterpart (Figure 1B, right panel). The sequential scheme revealed that the combinatorial treatment was the most effective strategy in impairing the growth of cetuximab-persister cells (Figure 1C,D).

### 2.2. Concomitant Treatment with Cetuximab and VitC Impairs CRC Cell Growth and Delays Emergence of Acquired Resistance in 2D and 3D in Vitro Models

**Figure 2:**
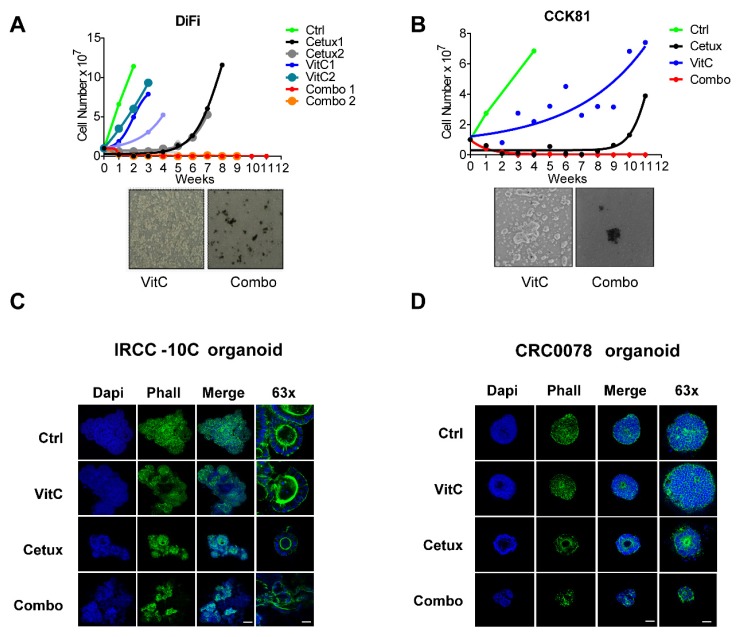
Combinatorial treatment hijacks the emergence of acquired resistance in preclinical CRC models in vitro. (**A**,**B**) Top part: Time-To-Progression assay performed in (A) DiFi and (B) CCK81 CRC cell lines. The effect of the indicated treatments on cell proliferation was assessed over time by cell counting. Nonlinear fit with exponential growth curve (Graphpad Prism) was applied to data points to show growth kinetics. Biological duplicates are shown for treatments on DiFi cells. Bottom part: bright-field microscopy images of DiFi and CCK81 cell lines treated with VitC alone and in combination with cetuximab were taken at the end of the experiment. (**C**) *RAS/BRAF* wild-type (wt) cetuximab-sensitive organoids (IRCC-10C-XO) were treated with VitC, cetuximab, and combinatorial treatment. At the end of the treatment schedule, organoids were stained with DAPI (blue) and phalloidin (Phall, green) in order to assess their 3D cellular structure. Representative images of organoids are shown for each condition (*n* = 3). Scale bar 100 μm (10 μm, 63×). (**D**) *RAS/BRAF* wt cetuximab-sensitive organoids obtained from a second independent patient (CRC0078) were treated with VitC, cetuximab, and combinatorial treatment and then stained with DAPI (blue) and phalloidin (Phall, green) in order to assess their 3D cellular structure. Representative images of organoids are shown for each condition (*n* = 3). Scale bar 50 μm (25 μm, 63×). Abbreviations and drug concentrations: Ctrl: control; VitC: Vitamin C (1 mM); Cetux: cetuximab (50 µg/mL); Combo: combination of VitC and cetuximab.

Next, we measured the time required for the cells to regain proliferative capabilities, compared with the parental counterpart in the absence of the drugs [4]. To this end we treated *RAS/BRAF* wt CRC cell models with VitC, cetuximab, or a combination of the two drugs and continuously monitored cell growth (Figure 2A,B). VitC marginally affected proliferation in the early phase, but the cells rapidly returned to the initial proliferation rates. Cetuximab-treated cells responded more prominently to the treatment but developed resistance within a few weeks. Notably, when cells were concomitantly treated with VitC plus cetuximab (Combo), the resistant population was apparently eradicated (Figure 2A,B). We monitored the plates for several months and no evidence of resistant colonies was observed (Figure 2A,B). At the end of the experiment, only cell debris was visible at bright-field microscopy (Figure 2A,B, lower panels). Comparable results were also observed in other cetuximab-sensitive CRC cell models treated with combination of VitC and cetuximab (Figure S2).

We extended the analysis to two independent patient-derived *RAS/BRAF* wt cetuximab-sensitive CRC organoids (IRCC-10C and CRC0078) and challenged them with the same treatments previously used in the 2D assays. In this setting, VitC did not affect organoid growth, while cetuximab was effective in restricting organoid size. Combinatorial treatment led to severe disassembly of organoid structure, as shown by immunostaining of cytoskeletal elements (Figure 2C,D).

### 2.3. Addition of VitC to Anti-EGFR Therapy Delays the Emergence of Acquired Resistance in Cetuximab-Sensitive Patient-Derived Xenograft Models

**Figure 3:**
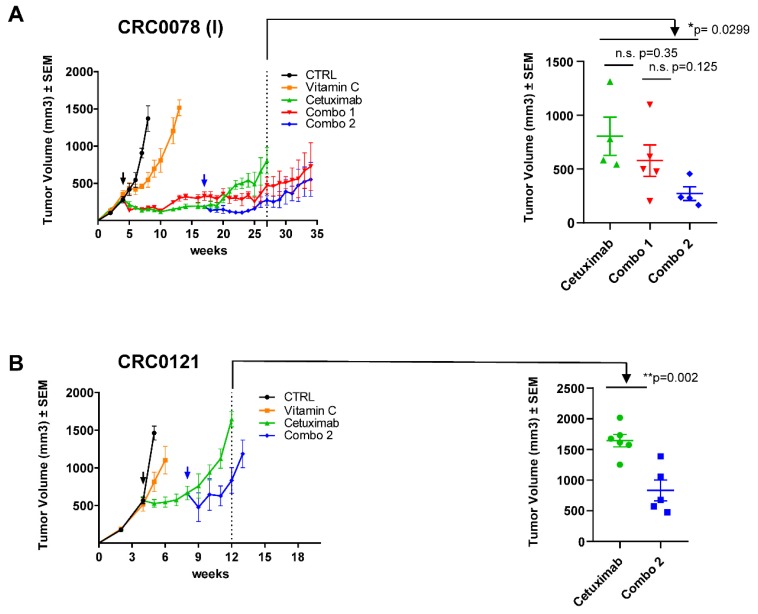
Combinatorial treatment delays the emergence of acquired resistance in cetuximab-sensitive patient-derived xenografts. (**A**) Left panel: a cetuximab-sensitive CRC PDX (CRC0078) was expanded to create four cohorts. When tumors reached around 300 mm^3^ in volume mice were randomized (black arrow) and treated with vehicle, VitC (4 g/kg, intraperitoneal injection), cetuximab (10 mg/kg, intraperitoneal injection), or their combination (Combo 1, red curve). A delayed combinatorial treatment called “Combo 2” (blue arrow) was initiated after 13 weeks of cetuximab treatment to intercept tumors in a drug-tolerant condition. Right panel: scatter plot showing comparison and statistical significance between mice treated with cetuximab, Combo 1, and Combo 2. (**B**) A second cetuximab-sensitive CRC PDX model (CRC0121) was expanded to create four cohorts. When tumors reached around 500 mm^3^ in volume mice were randomized (black arrow) and treated with vehicle, VitC (4 g/kg, intraperitoneal injection), cetuximab (10 mg/kg, intraperitoneal injection). A delayed combinatorial treatment (blue arrow, Combo 2) was initiated after 4 weeks of cetuximab treatment to intercept tumors in a drug-tolerant condition. Right panel: scatter plot showing comparison and statistical significance between mice treated with cetuximab and Combo 2. Error bars represent SEM. Statistical significance: n.s., not significant; *p < 0.05; **p < 0.01; (two-tailed unpaired Student’s *t*-test).

To further assess the potential relevance of the combinatorial therapy, we performed in vivo experiments, by treating *RAS/BRAF* wt CRC patient-derived-xenograft (PDX) models sensitive to anti-EGFR antibodies. Tumors were allowed to fully establish and then were challenged with VitC, cetuximab, or their combination (Figure 3A). VitC treatment had modest activity and slightly delayed tumor growth, while cetuximab treatment consistently reduced tumor size (Figure 3A). After few months of treatment, mice treated with cetuximab alone relapsed. Tumor growth eventually resumed also in mice that received combinatorial treatment (Combo 1, red curve), although this occurred later than in animals treated with cetuximab as a monotherapy (Figure 3A).

We speculated that prolonged treatment with VitC ab initio could activate compensatory mechanisms limiting its efficacy in vivo. We therefore aimed at intercepting EGFR-treated tumors in a drug-tolerant state. To test this, we initiated concomitant VitC dosing (Combo 2, blue curve) a few weeks after treatment with cetuximab had started. This approach significantly reduced tumor volume respect to cetuximab-monotherapy and delayed the emergence of resistance (Figure 3A,B). Remarkably, combinatorial treatment was effective even when cetuximab was delivered to larger tumors (500 vs. 300 mm^3^ in size), in two different PDX models (Figure S3 and Figure 3B). Overall, treatments were well tolerated. Mice were constantly monitored during the experimental procedures and no additional signs of stress were detected in any of the treatment arms.

### 2.4. Cetuximab as A Single Agent and in Combination with VitC Impairs Glucose Metabolism

To elucidate the single contribution of VitC and cetuximab treatment to the phenotypes observed above, and to identify the mechanisms underlying the effects of these treatments, we performed proteomic and metabolic analysis in CRC cells treated with either agents alone or their combination.

In a SILAC-based comparative assay, CRC cells were treated at different time points (4 and 24 h) in the presence of isotope-labelled amino acids. Upon incorporation of the labelled amino acids, equal amounts of protein extracts from treated (Heavy labelled) and untreated (Light labelled) cells were combined and subjected to mass spectrometry analysis.

Hierarchical clustering analysis of differentially expressed proteins (*p* < 0.05 and FDR < 0.05) revealed modulation of proteins involved in multiple biological pathways.

**Figure 4:**
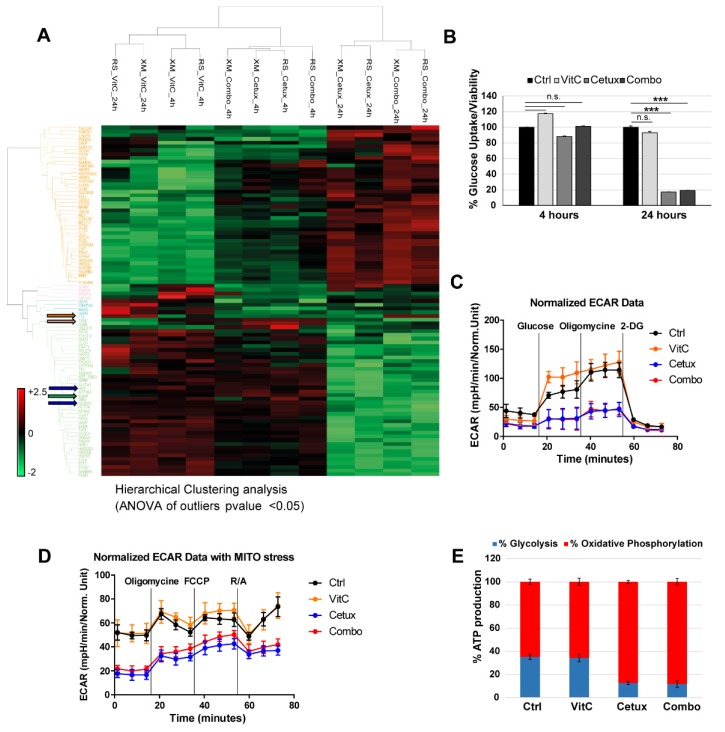
Proteomic and metabolic analysis of CRC cells treated with VitC or cetuximab as single agents or in combination. (**A**) Hierarchical clustering (HCL) of the proteins differentially expressed (ANOVA *p*-value < 0.05) in two independent batches of DiFi cells (RS and XM) treated for 4 and 24 h with VitC (1mM), cetuximab (50 μg/mL), or the combination. The heat map shows fold change in protein abundance compared with untreated cells. Blue arrows indicate SLC2A1 (GLUT1, Glucose-Transporter 1) and HK2 (Hexokinase2) proteins; green arrow indicates EGFR; orange and pink arrows indicate FT (Ferritin) and TFRC (Transferrin Receptor), respectively. (**B**) DiFi cells were treated for 4 and 24 h with the indicated drugs and glucose uptake was measured. One representative of three independent experiment performed each with technical triplicates is shown; error bars represent ± SD. (**C**) DiFi cells were treated for 24 h with VitC (1 mM), cetuximab (50 µg/mL), or their combination and Seahorse XF96 Extracellular Flux Analyzer was used to measure ExtraCellular Acidification Rate (ECAR) and (**D**) ECAR followed by a Cell MITO Stress Test. Continuous values normalized to micrograms of proteins are shown. Results are reported as mean ± SD of one representative of three independent experiments performed with at least three technical replicates each. (**E**) ATP production was measured by Seahorse XF Real-Time ATP rate assays after 24 h of treatment with the indicated drugs and source of ATP (glycolysis or oxidative phosphorylation) is shown as percentage of total ATP. R/A: Rotenone/Antimycin.

Cetuximab highly affected protein levels in combo-treated cells, as noted by co-clustering of the two treatments at 4 and 24 h (Figure 4A). As expected, cetuximab treatment determined downregulation of the EGFR pathway (Figure 4A, green arrow and Figure S4) and, interestingly, a more marked effect was observed in combo-treated cells, where a rebound reactivation of pERK was not evident as in cetuximab-treated cells, suggesting that combinatorial treatment might shut off or delay possible recovery mechanisms in cells (Figure S4). As it is known that EGFR plays a pivotal role in metabolism of lung and colorectal cancer [23,24], we investigated how cetuximab could affect metabolism in CRC cells and if any differential pathway could be activated in combination with VitC. We found that EGFR blockade modulates proteins involved in glucose, nucleotide, amino acid, lipid, and fatty acid metabolism (Figure 4A and Table S1). In particular, the central carbon metabolism was impaired through downregulation of the glucose transporter GLUT-1 (SLC2A1), and consequent inhibition of glucose uptake (Figure 4B), and of the glycolysis-initiating enzyme Hexokinase-2 (HK-2) (Figure 4A and Figure S5, Table S1). To functionally address how cetuximab, alone or in combination with VitC, affects glycolysis in our models, we exploited the Seahorse XF96 Extracellular Flux Analyzer to perform an ExtraCellular Acidification Rate assay (ECAR). We found that cetuximab, alone or in combination with VitC, impairs glycolytic function in DiFi cells (Figure 4C). When the same assay was performed in concomitance with a Cell MITO Stress test, we observed that, contrary to control and VitC-treated cells, cetuximab and combo-treated cells were unable to switch to glycolytic metabolism upon inhibition of the electron transport chain (ETC) complex (obtained by a combination of Rotenone/Antimycin A, complex I, and III inhibitor, respectively) (Figure 4D).

These data further confirm that cetuximab, alone or in combination with VitC, impairs glucose uptake and metabolism.

Proteomic analysis also suggested a switch from glycolysis to oxidative phosphorylation (OXPHOS) metabolism in cetuximab and combo-treated cells, as highlighted by downregulation of lactate dehydrogenase enzyme (LDH-A and LDH-B subunits), upregulation of pyruvate dehydrogenase subunits (PDHA1 and PDHB), and increased expression of respiratory enzymes associated to the ETC complex III (Table S1 and Figure S6A,B). Consistently, after 24 h of treatment with cetuximab or combo, total ATP levels were significantly decreased (Figure S7) and residual ATP was mainly originating from OXPHOS (Figure 4E).

Collectively, these data show that, at least at early timepoints, cetuximab and combo-treated cells are equally impaired at a metabolic level and this prompted us to look for other differentially activated proteins when comparing cetuximab versus combo-treated cells. We found that proteins related to iron metabolism such as ferritin (FT, orange arrow) and transferrin receptor (TFRC, pink arrow) were respectively up and downregulated in VitC and combo-treated cells at 24 h, thus confirming the involvement of VitC in perturbating iron metabolism (Figure 4A) [25].

These data altogether indicate that in our models EGFR blockade induces inhibition of glycolysis while in parallel VitC causes perturbation of iron metabolism, suggesting that the biological effects observed in combo-treated preclinical models might be due to an additive metabolic synthetic lethal effect exerted by the concomitant use of the two drugs.

### 2.5. Vitamin C Alters Iron Homeostasis and Triggers Ferroptosis in CRC Cells

The observation that cetuximab plus VitC treatment leads to cell death in CRC models, while at the same time influencing oxidative stress and iron metabolism, reminded us of ferroptosis, a recently described form of cell death that is iron- and reactive oxygen species (ROS)-dependent [26]. Ferroptosis involves accumulation of ‘free’ iron, which causes oxidative stress through Fenton catalysis, depletion of the antioxidant glutathione and accumulation of lipid oxidative damage, leading to cell membrane denaturation [27,28]. We indeed explored whether VitC is able to sustain these three main hallmarks of ferroptosis [29].

**Figure 5:**
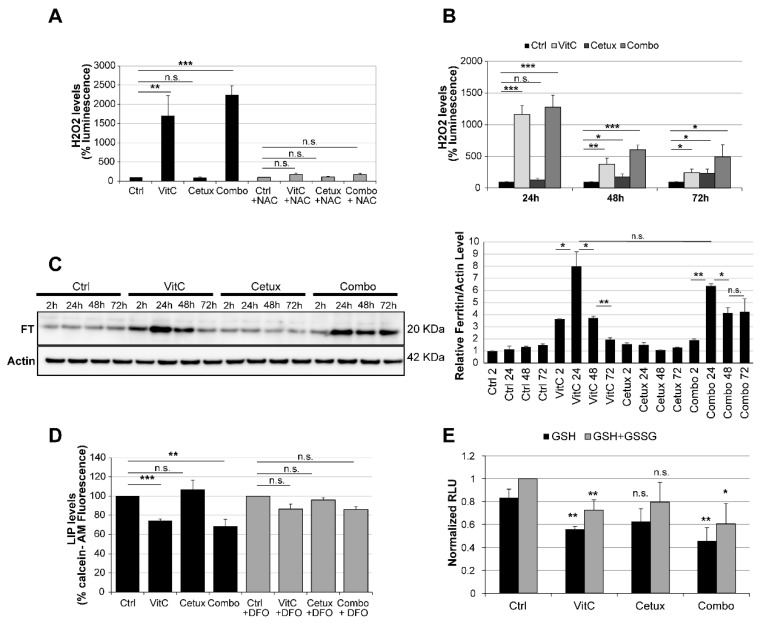
VitC-mediated ROS production triggers ferroptosis in CRC cells. (**A**) DiFi cells were treated as indicated for 4 h and ROS levels were measured; N-acetyl cysteine (NAC, 10 mM) was used as a control to rescue ROS production in drug treated cells. See also Figure S8 for results in C75 and CCK81 CRC cells. (**B**) DiFi were treated as indicated and ROS levels were measured after 24, 48, and 72 h. Results in (A) and (B) are representative of at least three independent experiments; results are normalized to relative controls and error bars represent SD. (**C**) Cells were treated as indicated and total protein lysates were analyzed for ferritin (FT) expression. Actin was used as a loading control. Right panel: Western blot quantification analysis was performed by the ImageJ software. (**D**) Cells were treated for 3 h with VitC, cetuximab, or the combination and labile iron pool (LIP) levels were measured by a calcein-AM method. LIP levels are inversely correlated with calcein fluorescence, indicating in this experiment that LIP levels are increased in cells treated with VitC or combo. Deferoxamine (DFO) (200 μM) rescues the increase in LIP at levels comparable to those of the control. (**E**) DiFi cells (12,000 cells/well) were plated in 96-well white walled plate and incubated overnight for their attachment to the plate surface. Cells were then treated for 24 h with VitC (1 mM), cetuximab (50 µg/mL), or their combination. The GSH/GSSG levels were assessed through GSH/GSSG-Glo™ Assay (Promega™) following manufacturer protocol. Data were normalized with respect to total GSH/GSSG in untreated cells. Statistical significance for each treatment was calculated with respect to GSH or total GSH/GSSG in untreated cells. Bars are the average of three independent experiments. Error bars represent SD. Statistical significance: n.s., not significant; *p < 0.05; **p < 0.01; ***p < 0.001 (two-tailed unpaired Student’s *t*-test).

We found that VitC as a single agent or in combination with cetuximab, triggered an increase of ROS production, and this effect was blunted by administration of the antioxidant N-Acetyl Cysteine (NAC) in different CRC cell lines (Figure 5A and Figure S8). Notably, in a time course experiment, cetuximab led to a less intense but progressively stronger ROS upregulation (Figure 5B), likely due to increased reliance on OXPHOS metabolism. Importantly, the addition of cetuximab prevented the rapid decay of ROS production observed after VitC treatment and enabled sustained ROS persistence in combo-treated cells (Figure 5B). Analogous results were obtained when we mimicked cetuximab effects by a RNA interference assay. Upon EGFR siRNA-mediated downregulation, we observed slightly increased levels of ROS at 24 and 48 h, which maintained higher ROS levels in combo-treated cells with respect to cells treated with the single VitC agent (Figure S9).

The levels of FT, a protein involved in iron storage, rapidly increased upon VitC treatment and then progressively declined, while remaining consistently elevated in combo-treated cells (Figure 5C). Increased levels of ROS can promote FT chemical reduction and release of iron in the cytoplasm [30,31]. Increased levels of labile iron pool (LIP) were indeed detected upon treatment with VitC, alone and in combination with cetuximab and this effect could be rescued by deferoxamine (DFO), an iron-chelating agent that binds free iron in a stable complex (Figure 5D). In parallel, we assessed levels of glutathione, a potent antioxidant that works also as a cofactor for enzymes involved in ferroptosis [28], in cells treated with VitC, cetuximab, and their combination and found that, after 24 h, VitC, alone or in combo, was able to significantly decrease levels of reduced and total glutathione (Figure 5E), suggesting that VitC treatment can acutely trigger oxidative stress leading to ferroptosis. In addition, we observed that lipid peroxidation was increased by the addition of VitC in CRC cells (Figure S10A) and that addition of ferrostatin-1 (FRS-1), a lipid ROS scavenger that prevents oxidized lipid accumulation and ferroptosis [32], significantly reduced lipid peroxidation in combo-treated cells (Figure S10B).

**Figure 6:**
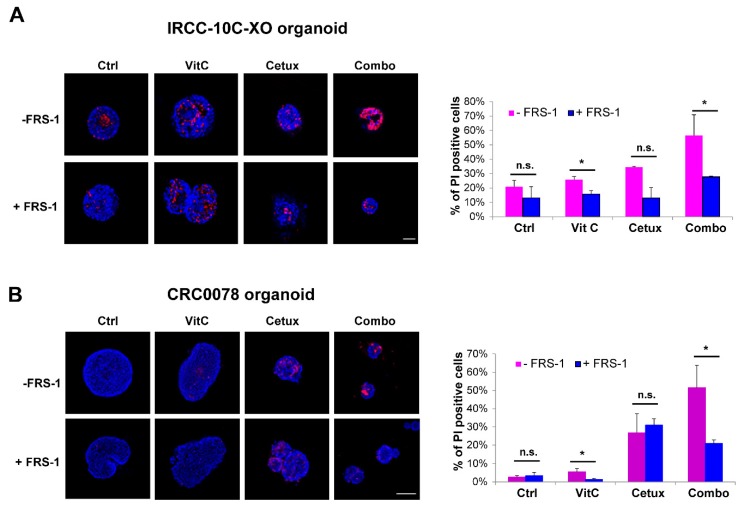
Combinatorial treatment increases lipid membrane damage, which is rescued by ferrostatin (FRS-1) treatment. (**A**,**B**) IRCC-10C-XO (A) and CRC0078 (B) organoids were treated with VitC, cetuximab, and combinatorial treatment for 2 weeks and then stained with HOECHST (blue) and PI (red) to assess the levels of membrane damage and cell death. FRS-1 (2 μM) was used to rescue the effects dependent on lipid peroxide toxicity. Representative images of organoids are shown for each condition (*n* = 3). Scale bar: 50 μM (IRCC-10C-XO) and 100 μM (CRC0078). Quantification with ImageJ software is shown in the right panel. Treatments: Ctrl, control media; VitC, 1 mM; Cetux, 50 μg/mL; Combo, VitC 1 mM plus Cetux 50 μg/mL. Error bars represent SD. Statistical significance: n.s., not significant; *p < 0.05; (two-tailed unpaired Student’s *t*-test).

To further corroborate this evidence, we tested two independent CRC *RAS/BRAF* wt organoid models. Increased levels of propidium iodide (PI) were found in organoids, especially in combo-treated ones, suggesting augmented levels of membrane damage that was rescued by the addition of FRS-1 (Figure 6A,B).

**Figure 7:**
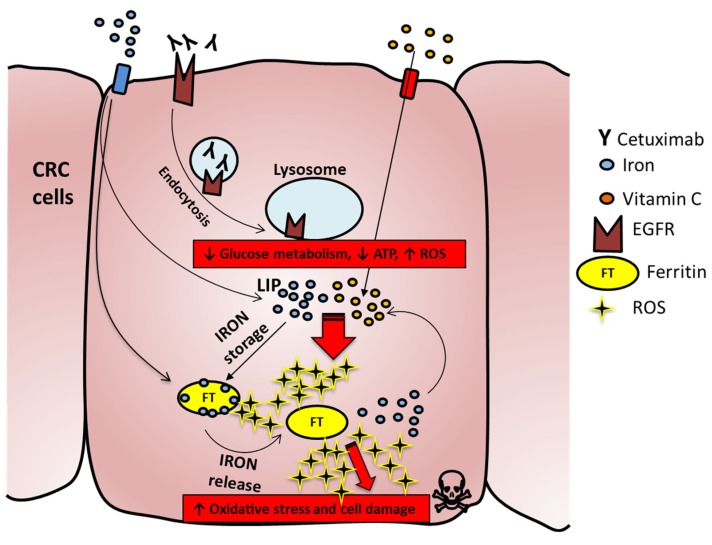
Proposed mechanism for increased ROS production in combo-treated cells. Iron enters in the cell and in part generates the labile iron pool (LIP), while a part is stored in the ferritin complex (FT). Cetuximab binds to EGFR and promotes the EGFR pathway and metabolic downregulation; VitC enters in CRC cells and triggers, by interacting with LIP, ROS formation, which in turn can chemically reduce FT and promote iron release. The latter will react with VitC, which is continuously provided to cells and will foster increased ROS production with consequent membrane damage and cell death.

Collectively, the above findings depict a potential model in which on one side anti-EGFR treatment induces downregulation of glycolysis and reduction of ATP levels, thus making cancer cells more susceptible to the oxidative stress induced by VitC. In parallel, addition of VitC triggers ferroptosis by increasing the amount of free iron involved in intracellular redox reactions (LIP), that in turn leads to increase of FT levels [33] and augments ROS production fueled by continuous VitC administration and exacerbated by concomitant cetuximab provision (Figure 7). Indeed, combinatorial treatment of pharmacological VitC with cetuximab exerts an unrecoverable metabolic synthetic lethal effect, ultimately leading to cancer cell death (Figure 7).

## 3. Discussion

Acquired resistance in CRC is a fait accompli [2]. A transient benefit from treatment with anti-EGFR targeted therapies initially occurs due to bulk killing of drug-sensitive *RAS/BRAF* wt cells and consequent measurable tumor volume reduction. The surviving clones (i.e., persister cells) are characterized by a drug-tolerant state that could rely on different mechanisms, either genetic or nongenetic (i.e., transcriptional, epigenetic, metabolic) [9]. Prolonged exposure to the drug progressively fuels the emergence of a heterogeneous resistant population that leads eventually to the therapeutic failure. We reasoned that targeting the tumor in a drug-tolerance state and exploiting transient metabolic vulnerabilities might represent an effective strategy to eradicate persister clones, thus hampering the development of acquired resistance to the therapy. VitC is an organic molecule with proved pro-oxidant effects when administered at high doses [13]; moreover, it has been shown that VitC might eradicate *KRAS/BRAF* mutant clones, which in our previous studies emerged as major mechanisms of relapse to anti-EGFR therapy [34].

We therefore reasoned that an impaired metabolic state, determined by EGFR blockade, together with oxidative stress imposed by VitC treatment, could improve the efficacy of anti-EGFR antibodies in CRCs. To our knowledge, this is the first study showing that VitC in combination with cetuximab restricts the emergence of acquired resistance in *RAS/BRAF* wt CRC. We focused on *RAS/BRAF* wt models to closely mimic the clinical setting where only CRC patients with this type of genetic profile are allowed to receive anti-EGFR treatment.

At a mechanistic level, we show that cetuximab severely impairs central carbon metabolism by inhibiting the glucose transporter and consequently glucose uptake. Interestingly, large-scale proteomic analysis and metabolic flux assays suggest also a switch to OXPHOS metabolism, which is consistent with previous work showing how cells attempting to survive in a nutrient-depleted environment enter a quiescence state [35].

The overall effect of EGFR blockade and VitC treatment is reminiscent of data showing that fasting regimens promote the anti-Warburg effect and reduce MAPK signaling, thus enhancing the effect of chemo and targeted therapies [36,37]. In line with this, we observed downregulation of EGFR downstream signaling in both cetuximab and combo-treated cells. Notably, the previously observed ERK rebound activation [34] was evident only in cetuximab monotherapy treated cells, but not in the combinatorial arm. This evidence suggests that while cells treated with VitC or cetuximab monotherapies might be able to recover through activation of defense or escape mechanisms, combinatorial treatment exerts a metabolic synthetic lethality effect that completely impinges on survival of persister cells.

We noted that the expression levels of proteins involved in iron homeostasis were perturbed by VitC treatment and confirmed that this could trigger massive production of ROS with consequent GSH depletion and membrane lipid damage. According to this experimental evidence in multiple patient-derived preclinical models, we suggest for the first time that pharmacological doses of VitC might trigger ferroptosis and that mobilization of iron pools by VitC could represent a promising strategy to induce ROS-mediated stress and cell death in CRC cells whose metabolism is impaired by targeted drug treatment [38].

As a functional readout, combinatorial treatment with cetuximab and VitC hampered the growth of CRC cells, ultimately preventing the emergence of acquired resistance to anti-EGFR targeted therapy in vitro. Organoid structure was impaired by combinatorial treatment and results in CRC PDXs suggest that the combination with VitC is superior to EGFR blockade alone also *in vivo*. Although we did not observe extensive tumor regression in mice, the effect was still relevant, considering that it was achieved by addition of a vitamin known to have little or no side effects in humans [18,20]. We acknowledge that we did not perform analysis of ferroptosis markers in PDXs, so further investigation should be accomplished to assess contribution of VitC to ferroptosis in these models, taking also into account that mice, unlike humans, are able to synthesize their own endogenous vitamin C.

Future preclinical studies are also warranted to test whether VitC addition to chemotherapy and EGFR-targeted antibodies could potentiate the efficacy and duration of recommended first-line regimens in CRC.

VitC is a known cofactor for several Fe^2+^ and α-ketoglutarate-dependent dioxygenases, including enzymes regulating histone and DNA methylation, such as ten-eleven translocation (TET) DNA hydroxylases. Indeed, it has been reported that VitC can act as an epigenetic remodeling agent on cancer cells [39,40], as well as on several non-neoplastic cell types, including immune cells [41,42]. The experimental models used in this work (cells, organoids, human tumors transplanted in immunocompromised animals) were not suited to assess the potential effect of VitC on modulating anticancer immune responses [43,44]. The activity might therefore be magnified in the presence of a fully competent immune system.

In conclusion, our data have the potential to be rapidly translated into clinical trials testing the effects of VitC in combination with EGFR-targeted monoclonal antibodies in metastatic CRC patients. Contrary to many drug combinations previously tested to curb the emergence of resistance to targeted therapies, the innovative treatment proposed here is expected to add no or limited toxicity to an already approved regimen, although we acknowledge that further clinical investigation for establishment of optimal dosing and scheduling is needed.

## 4. Methods

### 4.1. Cell Lines and Cell Authentication

DiFi cells were a kind gift from Dr. J. Baselga in November 2004 (Oncology Department of Vall d’Hebron University Hospital, Barcelona, Spain), and were described for the first time in [45]. DiFi is a KRAS/BRAF wt CRC cell line established from a familial adenomatous polyposis patient and is sensitive to anti-EGFR blockade due to EGFR gene amplification.

Regarding the other CRC RAS/BRAF wt cell lines described in this study, CCK81 were obtained from Health Science Research Resources Bank and C75 were obtained from ECACC and cultured according to supplier guidelines. IRCC-10A was established as described in the next section.

DiFi and IRCC-10A cell lines were cultured in DMEM/F12 medium; CCK81 cell line was cultured in MEM medium; C75 cell line was cultured in RPMI1640 medium. Culture media was supplemented with fetal bovine serum (10%), 2 mM L-glutamine, antibiotics (100 U/mL 1 penicillin and 100 mg/mL 1 streptomycin). Cell lines were grown in a 37 °C and 5% CO_2_ air incubator.

The genetic identity of cell lines was authenticated before performing experiments by PowerPlex^®^ 16 HS System (Promega, Madison, WI, USA), through short tandem repeats (STR) at 16 different loci (D5S818, D13S317, D7S820, D16S539, D21S11, vWA, TH01, TPOX, CSF1PO, D18S51, D3S1358, D8S1179, FGA, Penta D, Penta E, and amelogenin). Amplicons from multiplex PCRs were separated by capillary electrophoresis (3730 DNA Analyzer, Applied Biosystems, Foster City, CA, USA) and analyzed using GeneMapper v.3.7 software (Life Technologies, Carlsbad, CA, USA). All cell lines were also routinely tested for mycoplasma contamination with the PCR Mycoplasma Detection Kit (ABM, Richmond, BC, Canada).

### 4.2. Tumor Specimen and Patient-Derived Model Establishment

Patient tumor samples were obtained from patients treated by liver metastectomy at Niguarda Cancer Center (Milano, Italy) and Ospedale Mauriziano Umberto I (Torino, Italy). All patients provided informed written consent, samples were procured and the study was conducted under the approval of the local Ethical Committee of the institutions (for Niguarda Cancer Cencer: study 194/2010, n.22 date 24th February 2010, for Candiolo Cancer Center: PROFILING study, 001-IRCC-00IIS-10, 6.0 version, date 24th April 2015).

The study was conducted according to the provisions of the Declaration of Helsinki.

To generate PDXs (patient-derived xenografts), tumor specimens were subcutaneously implanted in 7-week-old NOD-SCID mice (Charles River Laboratory, Wilmington, MA, USA). Treatments were performed as described in the in vivo experiments section. The protocol was reviewed by the local Animal Welfare Body (OPBA) of the Candiolo Cancer Institute IRCCS (Candiolo, Italy) and authorized by Italian Ministry of Health (Licence Number 195/2015-PR and 154/2019-PR), according to the Italian Law on the protection of animals used for scientific purposes (DLgs 26/2014) which enforces the EU Directive 63/2010.

The two PDX models used in this work were derived from quadruple wt (*KRAS*, *NRAS*, *BRAF* and *PIK3CA*) cetuximab-sensitive liver metastasis of CRC patients (CRC0078 and CRC0121).

IRCC-10A is a tumor-derived 2D-growing primary cell line obtained from a metastasis located in the hepatic right lobe of a *KRAS* wt CRC patient (IRCC-10).

Briefly, the tumor sample was dissociated into single-cell suspension by mechanical dissociation using the gentleMACS Dissociator (Miltenyi Biotec, Bergisch Gladbach, Germany) and enzymatic degradation of the extracellular matrix using the Tumor Dissociation Kit (Miltenyi Biotec, Bergisch Gladbach, Germany) according to the manufacturer’s instructions. The cell suspension was then centrifuged three times at 1200 rpm for 5 min and washed with DMEM/F12 medium. Supernatants were removed and cell pellets were resuspended with DMEM/F12 medium containing 10% FBS. The cell suspensions were then filtered through a 70-μm cell strainer (Miltenyi Biotec, Bergisch Gladbach, Germany) and resuspended with culture medium DMEM-F12 containing 2 mmol/L l-glutamine, antibiotics (100 U/mL penicillin and 100 μg/mL streptomycin), 50 μg/mL gentamicin, and 10 μmol/L ROCK inhibitor Y-27632 (Selleck Chemicals Inc., Houston, TX, USA ) and cultured on collagen-coated dish (Corning, NY, USA) at 37  °C in 5% CO_2_.

IRCC-10C-XO and CRC0078-XO organoids were established from PDX models of liver metastases of cetuximab-sensitive *RAS/BRAF* wt CRC patients (named IRCC-10 and CRC0078 respectively). In particular, IRCC-10C-XO was derived from a liver metastatic nodule located in hepatic segment 2, different from the one from which the abovementioned cell line was derived (right hepatic lobe). Briefly, tumor specimens were initially smashed in fragments of 2–4 mm in size and then chopped with scalpel to further mechanically dissociate in small pieces. After centrifugation, the pellet was washed and centrifuged three times with PBS. At the end of the washing phase, the pellet was resuspended in Basement Membrane Extract (BME; Cultrex BME RGF type 2, Trevigen, Gaithersburg, MD, USA) and 60 μL of organoids-BME suspension was dispensed into the center of each well of a 48-well plate. Different densities of tumor cells were plated and left to solidify before tumor organoid medium was added and tumor cells were incubated at 37 °C and 5% CO_2_ air incubator. The composition of Tumor Organoid medium is as follows: DMEM/F12 + Hepes medium supplemented with antibiotics, 1 × Primocin (InvivoGen, San Diego, CA, USA), 1% GlutaMax (Invitrogen, Carlsbad, CA, USA), 1 × B27 supplement (Invitrogen, Carlsbad, CA, USA), 1.25 mM N-acetyl-cysteine (Sigma Aldrich, St. Louis, MO, USA), 10 mM nicotinamide (Sigma Aldrich, (Sigma Aldrich, St. Louis, MO, USA), 50 ng/mL human EGF (PeproTech), 100 ng/mL R-spondin (R&D, Minneapolis, MN, USA), 100 ng/mL Noggin (PeproTech, London, UK), 10 nM gastrin (Sigma Aldrich, St. Louis, MO, USA), 500 nM TGFb type I receptor inhibitor A83-01 (Sigma Aldrich, St. Louis, MO, USA), 10 uM p38 MAPK inhibitor SB202190 (Sigma Aldrich, St. Louis, MO, USA), and 10 nM prostaglandin E2 (Tocris, Bristol, UK). Fresh medium was replaced every 2–3 days. Outgrowing organoids were passaged every 10–15 days after mechanical and enzymatic disruption.

The treatments of established organoids were performed in DMEM/F12 medium supplemented with 2 mM L-glutamine, antibiotics (100 U/mL penicillin and 100 mg/mL streptomycin) at 37 °C and 5% CO_2_.

### 4.3. Drugs and Reagents

Cetuximab was provided by Merck (Darmstadt, Germany). VitC solution (+)-Sodium L-ascorbate—Sigma^TM^ was prepared weekly by resuspending the powder in PBS for in vitro experiments. For animal experiments VitC was prepared weekly by resuspending the powder in sterile water and storing the solution at 4 °C.

### 4.4. Drug Proliferation Assay

Long-term assay: DiFi cells were seeded (25,000 cells/well) in 24-well plate and treated with VitC (1 mM), cetuximab (50 μg/mL), or their combination. When cells seeded in the control wells reached confluence (10 days), all wells were fixed with paraformaldehyde and stained with crystal violet.

Short-term assay after generation of cetuximab-persister cells: DiFi cells previously treated for 2 weeks with cetuximab (100 μg/mL) or control medium were detached, counted, seeded in 96-well plates (3 × 10^3^ cells/well in technical triplicate), and incubated overnight for attachment. The day after they were treated with increasing amount of cetuximab or VitC and after 6 days viability was measured by CellTiter-Glo^®^ Luminescent Cell Viability Assay (Promega, Madison, WI, USA) according to the manufacturer’s protocol. Luminescence was measured using a plate-reading luminometer (TECAN Spark 10M, Männedorf, Switzerland); the resulting data were normalized to untreated cells and nonlinear fit analysis was performed with the Prism Graphpad software v. 8.

### 4.5. Time-To-Progression (TTP) Assay

For the TTP long-term assay, 10 million DiFi cells were plated in their respective growth media and treated from the day after with cetuximab (50 μg/mL), VitC (1 mM), or the combination of the two. All cells were counted at least once per week. Count as 0 represent time points in which cells were dead or too few and only medium and drug refreshments were done.

### 4.6. Western Blotting Analysis

Total cellular proteins were extracted by solubilizing the cells in EB buffer (50 mmol/L Hepes pH 7.4, 150 mmol/L NaCl, 1% Triton X-100, 10% glycerol, 5 mmol/L EDTA, 2 mmol/L EGTA; all reagents were from Sigma-Aldrich (St. Louis, MO, USA), in the presence of 1 mmol/L sodium orthovanadate, 100 mmol/L sodium fluoride, and a mixture of protease inhibitors. Extracts were clarified by centrifugation and normalized with the BCA Protein Assay Reagent Kit (Thermo Fisher Scientific, Waltham, MA, USA). Western blot detection was performed with enhanced chemiluminescence system (GE Healthcare, Chicago, IL, USA) and peroxidase conjugated secondary antibodies (Amersham, Little Chalfon, UK). The following primary antibodies were used for Western blotting (all from Cell Signaling Technology, Danvers, MA, USA, except where indicated): anti-phospho-p44/42 ERK (Thr202/Tyr204; 1:1000); anti-p44/42 ERK (1:1000); anti-phospho EGFR (Tyr1068) (1: 1000); anti-EGFR (EnzoLifeSciences, Farmingdale, NY, USA) (1:1000); anti-ferritin light chain (Abcam, Cambridge, UK) (1:1000); anti-Actin (Santa Cruz Biotechnology, Dallas, TX, USA) (1:1000).

### 4.7. Immunofluorescence

Organoids embedded in BME (Cultrex^®^ Basement Membrane Matrix BME, Trevigen, Gaithersburg, MD, USA) were grown in 8-well chamber slides, in DMEM/F12 10% FBS and treated with the indicated treatments. Drug was refreshed every 4 days. After 14–21 days, organoids were fixed in 4% paraformaldehyde for 30 min at room temperature (RT) and permeabilized with 0.5% Triton-X100 in PBS for 30 min RT. Nuclei were stained with DAPI. F-actin was stained with Alexa Fluor^®^ 488 or Alexa Fluor^®^ 647 Phalloidin (50 μg/mL). Slides were then mounted using the fluorescence mounting medium (Dako, Glostrup, Denmark) and analyzed using a confocal laser-scanning microscope (TCS SPE II; Leica, Wetzlar, Germany).

We took advantage also of live/dead staining to assess cell death in organoids by fluorescence microscopy. Matrigel was disrupted mechanically with a pipette tip and organoids were transferred to an Eppendorf tube. Organoids were stained in DMEM/F12 medium with 100 μg/mL Hoechst 33342 (Sigma Aldrich, St. Louis, MO, USA) and 100 μg/mL PI (Sigma Aldrich, St. Louis, MO, USA). After 30 min of incubation in the dark, organoids viability was observed by fluorescent microscopy (Axio Vert.A1, Zeiss, Oberkochen, Germany).

### 4.8. In Vivo Experiments

The PDX models used in this study were derived from patients (CRC0078 and CRC0121) carrying a quadruple wild-type (*KRAS*, *NRAS*, *BRAF*, and *PIK3CA*) colorectal tumor. Established tumors (average volume 300 or 500 mm^3^, as indicated) were treated with the following regimens, either single-agent or in combination: VitC (Sigma, 4 g/kg, intraperitoneal, daily-5 days per week), cetuximab (Merck, 10 mg/kg, intraperitoneal, twice weekly). Tumor size was evaluated every week by caliper measurements, and the approximate volume of the mass was calculated using the formula 4/3 π (d/2)2 D/2, where d is the minor tumor axis and D is the major tumor axis. Mice were kept under the constant supervision of veterinary personnel and animal procedures were approved by the Ethical Commission of the Candiolo Cancer Institute and by the Italian Ministry of Health (Licence Number 195/2015-PR and 154/2019-PR).

### 4.9. SILAC (Stable Isotope Labeling by Amino acids in Cell culture) Analysis

For SILAC analysis, two independent batches of DiFi cells (RS and XM Difi cells) were grown in reconstituted DMEM/F12 medium containing either “light” [^12^C6, ^14^N4] arginine (referred to as Arg0) and [^12^C6, ^14^N2] lysine (referred to as Lys0) or “heavy” [^13^C6, ^15^N4] arginine (referred to as Arg10) and [^13^C6, ^15^N2] lysine (referred to as Lys8) stable isotope labelled amino acids (Thermo Fisher Scientific, Waltham, MA, USA). After at least six passages to allow the incorporation of the isotopes into the cellular proteome, both RS and XM DiFi cell populations, labelled with heavy isotopes, were treated with VitC (1mM), cetuximab (50 μg/mL), and their combination for 4 and 24 h. The nontreated control, labelled with light isotopes, was cultured in parallel with the treated cells. After verification of the incorporation efficiency, DiFi cells pellet was solubilized in 8 M urea, 0.1 M Tris/HCl pH 8.5 (UA buffer) and equal amounts of protein extracts from treated and untreated labelled cells were combined 1:1, then subjected to mass spectrometry analysis.

### 4.10. Mass Spectrometry Analysis 

About 50 µg of proteins for each sample were reduced by TCEP, alkylated by chloroacetamide, and digested overnight by Lys-C and trypsin [46]. Derived peptides were desalted on StageTip C18 [47].

Samples were analyzed in duplicate on a LC–ESI–MS-MS quadruple Orbitrap QExactive-HF mass spectrometer (Thermo Fisher Scientific, Waltham, MA, USA). Peptides separation was achieved on a linear gradient from 95% solvent A (2% ACN, 0.1% formic acid) to 50% solvent B (80% acetonitrile, 0.1% formic acid) over 120 min, and from 50% to 100% solvent B in 2 min at a constant flow rate of 0.25 µL/min on UHPLC Easy-nLC 1000 (Thermo Fischer Scientific, Waltham, MA, USA) connected to a 23 cm fused-silica emitter of 75 µm inner diameter (New Objective, Inc. Woburn, MA, USA), packed in-house with ReproSil-Pur C18-AQ 1.9 µm beads (Dr Maisch Gmbh, Ammerbuch, Germany) using a high-pressure bomb loader (Proxeon, Odense, Denmark). MS data were acquired using a data-dependent top 15 method for HCD fragmentation. Survey full scan MS spectra (300–1650 Th) were acquired in the Orbitrap with 60,000 resolution, AGC target 3e6, IT 20 ms. For HCD spectra, resolution was set to 15,000 at m/z 200, AGC target 1e5, IT 80 ms. For identification and quantitation, Raw MS files were processed with MaxQuant software (1.5.2.8, https://www.maxquant.org) making use of the Andromeda search engine [48]. MS/MS peak lists were searched against the database uniprot_cp_human_2015_03, in which multiplicity was set as 2, trypsin specificity was used with up to two missed cleavages allowed. Cysteine carbamidomethyl was used as fixed modification, methionine oxidation and protein N-terminal acetylation as variable modifications. The peptides and protein false discovery rates (FDR) were set to 0.01; the minimal length required for a peptide was six amino acids; a minimum of two peptides, of which one unique was required for protein identification.

Normalized H/L ratios of proteins identified were analyzed via Perseus (version 1.5.6.0, https://maxquant.net/perseus). *t*-test and ANOVA statistical analysis was performed applying FDR < 0.05 or *p*-value < 0.05 as reported.

### 4.11. Glucose Uptake Assay

Twelve thousand DiFi cells were plated in 80 uL of DMEM/F12 media on day 1. On day 2 cells were treated for 4 and 24 h with the indicated drugs and glucose uptake was measured with the Glucose Uptake-Glo Assay (Promega, Madison, WI, USA) after incubation for 10 min in glucose-deprived medium supplemented with 1 mM 2-Deoxy-D-Glucose (2DG). Glucose-uptake values were normalized on cell viability; experiments were repeated three times with technical triplicates.

### 4.12. Seahorse Metabolic Experiments

Real-time measurements of extracellular acidification rate (ECAR) were made using an XFe96 Extracellular Flux Analyzer (Agilent Technologies, Santa Clara, CA). ECAR was measured on 17,000 cells, pretreated with the indicated drugs, using Glycolysis Stress Test kit, in basal conditions and in response to glucose (10 mM), oligomycin (1 μM), and 2-Deoxy-D-glucose (2-DG, 50 mM). The MITO stress experiment was performed using MITO Stress Test Kit following the manufacturer’s protocol and standard concentrations of oligomycin (1 μM), carbonylcyanide-4- (trifluoromethoxy)-phenylhydrazone (FCCP, 1 μM), and Rotenone/Antimycin A (0.5 μM). All reagents were from Agilent (Santa Clara, CA, USA).

To evaluate the rate of adenosine triphosphate (ATP) production in living cells, distinguishing between the fractions of ATP that are produced from OXPHOS and glycolysis, Agilent Seahorse XF Real-Time ATP rate assay was used following the manufacturer’s instructions.

Results were analyzed in WAVE software (version 2.6, Agilent), normalized on cellular protein concentration (OD), and processed through the XF Cell MITO Stress Test Report, Glycolysis Stress Test Generator and ATP rate assay generator. Each experiment was repeated at least three independent times with technical triplicates.

### 4.13. H_2_O_2_ Detection Assay

DiFi cells were seeded in a 96-well white walled plate (12 × 10^3^ cells/well) in 60 μL of medium per well and incubated overnight for their attachment to the plate surface. ROS-Glo™ H_2_O_2_ Assay (Promega™) was exploited to assess H_2_O_2_ levels. Then, 20 µL of a medium 5× drug-containing solution and 20 µL of H_2_O_2_ substrate solution (the latter only in the last 4 h of the treatment) were added to each well, reaching a final volume of 100 µL. The cells were placed in a 37 °C and 5% CO_2_ air incubator for 4, 24, 48, and 72 h of treatment. At the end of the treatment, 100 µL of ROS-Glo™ Detection Solution was added to each well. The relative luminescence was measured using a plate-reading luminometer (TECAN Spark 10M instrument) after 20 min of incubation at room temperature. The resulting data were normalized to untreated cells.

N-acetyl-L-cysteine (NAC, Sigma Aldrich, St. Louis, MI, USA) was used at the concentration of 10 mM to perform the rescue experiments.

### 4.14. siRNA Screening

The siRNA targeting reagents were purchased from Dharmacon (Lafayette, CO, USA), as a SMARTpool of four distinct siRNA species targeting different sequences of the target transcript. Cell lines were grown and transfected with SMARTpool siRNAs (20 nmol/L) using RNAiMAX (Invitrogen Carlsbad, CA, USA) transfection reagents following manufacturer’s instructions in a 96-well white plate for ROS assay and in 6-well plates for Western blot analysis after 48 and 72 h post-transfection. AllStars (Qiagen, Hilden, Germany) nontargeting siRNA was used as negative control. For ROS assay, cells were treated the day after transfection.

### 4.15. Labile Iron Pool (LIP) Level Analysis

DiFi cells were plated in 96-well microplates for fluorescence assays and incubated overnight for their attachment to the plate surface. Calcein AM solution (C1359, Sigma Aldrich, St. Louis, MI, USA) was added to each well following manufacturer’s instructions and fluorescent levels were measured by TECAN Spark 10M instrument. LIP levels are inversely correlated with calcein fluorescence.

Deferoxamine mesylate (DFO, Sigma Aldrich, St. Louis, MI, USA) was supplemented at 200 μM to cells to perform the rescue experiment.

### 4.16. Membrane Lipid Peroxidation Detection Assay

Twenty million DiFi cells were plated in 15 cm Petri dishes (two dishes per treatment) and then treated for 24 h with VitC, cetuximab, or their combination. Lipid peroxidation was assessed using the OxiSelect™ TBARS Assay Kit (Cell Biolabs, San Diego, CA, USA). Cells were counted and an equal amount for each sample was resuspended in PBS containing 1× BHT. Cell suspensions were then sonicated. Whole homogenate was then processed following manufacturer protocol. Ferrostatin-1 (FRS-1, Sigma Aldrich, St. Louis, MI, USA) was used at the final concentration of 2 μM to perform the rescue experiment.

### 4.17. Statistical Analysis

Results were expressed as means ± standard error of the mean (SEM) or standard deviation (SD) as indicated in the legend. Statistical significance was evaluated by unpaired two-tailed Student’s *t*-test, using GraphPad Prism^®^ 8.00 (GraphPad Software, Inc., La Jolla, CA, USA). *p* < 0.05 was considered statistically significant.

## 5. Conclusions

EGFR-targeted therapies have shown limited efficacy in mCRC patients due to the emergence of acquired resistance. The identification of ‘non-toxic’ therapeutic combinations capable of restraining secondary resistance represents therefore an urgent clinical need. Here we provide the first evidence that concomitant cetuximab-induced metabolic rewiring of CRC cells and vitamin C-mediated oxidative stress might restrict the emergence of secondary resistance to EGFR blockade. These findings could pave the way to future clinical experimentations involving *RAS/BRAF* wt mCRC patients treated with anti-EGFR antibodies.

## Supplementary Materials

The following are available online at https://www.mdpi.com/2072-6694/12/3/685/s1, Figure S1: Effects of VitC treatment on cetuximab persister CRC cells, Figure S2: Combinatorial treatment of cetuximab and VitC induces cell death in CRC Cells, Figure S3: Combinatorial treatment with VitC significantly delays acquired resistance to cetuximab in CRC PDX models, Figure S4: Cetuximab alone or in combination with VitC downregulates the EGFR downstream pathway, Figure S5: Cetuximab alone or in combination with vitC downregulates proteins involved in glycolysis, Figure S6: Proteomic analysis reveals a switch towards OXPHOS metabolism by regulating levels of enzymes involved in pyruvate metabolism and mitochondrial respiration in cells treated with cetuximab or combo, Figure S7: ATP levels decrease in cetuximab and combo treated cells, Figure S8: VitC treatment triggers ROS production in CRC cells, Figure S9: EGFR siRNA mediated downregulation phenocopies cetuximab mediated ROS production, Figure S10: Vitamin C alone or in combination with cetuximab increases levels of lipid peroxidation in CRC cells, Table S1: List of candidate proteins obtained from SILAC-based proteomic analysis of DiFi cells treated at 4 and 24 h with VitC, cetuximab, or the combination.
