# Supplementary material for: Vitamin C Restricts the Emergence of Acquired Resistance to EGFR-Targeted Therapies in Colorectal Cancer
Source: Cancers (Basel). 2020 Mar 14;12(3):685. doi: 10.3390/cancers12030685 (PMC7140052; doi:10.3390/cancers12030685)
Supplement: Supplementary file 1 [file cancers-12-00685-s001.zip › cancers-735820-suppl/cancers-735820-suppl-Proofreading_SA final.docx]

Supplementary Materials: Vitamin C Restricts the Emergence of Acquired Resistance to EGFR-Targeted Therapies in Colorectal Cancer

Annalisa Lorenzato, Alessandro Magrì, Vittoria Matafora, Valentina Audrito, Pamela Arcella, Luca Lazzari, Monica Montone, Simona Lamba, Silvia Deaglio, Salvatore Siena, Andrea Bertotti, Livio Trusolino, Angela Bachi, Federica Di Nicolantonio, Alberto Bardelli and Sabrina Arena

**Figure S1.** Effects of VitC treatment on cetuximab-persister CRC cells. CCK81 cells were seeded (10000 cells/well) in 24-well plate for a long-term proliferation assay under treatment with VitC (1 mM), cetuximab (50 μg/ml) or their combination. After 10 days, all wells were fixed with paraformaldehyde and stained with crystal violet. Representative images from one of three independent experiments are shown.

**Figure S2.** Combinatorial treatment of cetuximab and VitC induces cell death in CRC cells. Bright-field microscopy of a primary cell line (IRCC-10A) derived from a cetuximab-sensitive *RAS/BRAF* wt CRC patient (IRCC-10) and of C75 CRC cell line treated with VitC alone or in combination with cetuximab.

**Figure S3.** Combinatorial treatment with VitC significantly delays acquired resistance to cetuximab in CRC PDX models**.** CRC0078 (second experiment): technical replicate of the first PDX model, where the treatment started with larger (500 mm^3^) tumors. For ethical reasons, only cetuximab and Combo 2 arms were performed. When tumors reached around 500 mm^3^ mice were randomized (black arrow) and treated with cetuximab. A delayed combinatorial treatment (blue arrow, combo 2) was initiated after 5 weeks of cetuximab treatment to intercept tumors in a drug-tolerant condition. Right panel: scatter plot showing comparison and statistical significance between mice treated with cetuximab and Combo 2. Error bars represent SEM. Statistical significance: **p* < 0.05 (two-tailed unpaired Student’s *t*-test).

**Figure S4.** Cetuximab, alone or in combination with VitC, downregulates EGFR downstream pathway. DiFi cells were treated with indicated agents for 2 h, 24 h and 48 h and EGFR effectors were assessed at a biochemical level; actin was used as a loading control. Treatments: Ctrl, control media; VitC, 1 mM; Cetux, 50 μg/mL; Combo, VitC 1 mM plus Cetux 50 μg/mL.

**Figure S5.** Cetuximab, alone or in combination with VitC, downregulates proteins involved in glycolysis. HK2 and GLUT1 protein levels obtained from SILAC analysis. Statistical significance: n.s., not significant; ***p* < 0.01; ****p* < 0.001 (two-tailed unpaired Student’s *t*-test).

**A**

**B**

**Figure S6.**  Proteomic analysis reveals a switch towards OXPHOS metabolism by regulating levels of enzymes involved in pyruvate metabolism and mitochondrial respiration in cells treated with cetuximab or Combo. SILAC analysis performed in two independent batches of DiFi cells treated at 4 and 24 hours with VitC, cetuximab or the combination shows (**A**), downregulation of Lactate Dehydrogenase enzyme (LDH-A and LDH-B subunits) and upregulation of the Pyruvate Dehydrogenase enzyme (PDH α- and β-subunits); (**B**), upregulation of mitochondrial respiratory chain proteins in cells treated with cetuximab or Combo (see also Table S1).

**Figure S7.** ATP levels decrease in cetuximab and combo-treated cells. DiFi cells were treated with cetuximab, VitC or the combination for 24 h. ATP content was measured by Seahorse XF. One representative of three independent experiments is shown. Error bars represent SD. Statistical significance is calculated respect to control cells: n.s., not significant; ****p* < 0.001 (two-tailed unpaired Student’s *t*-test).

**Figure S8.** VitC treatment triggers ROS production in CRC cells. C75 cells (top) and CCK81 cells (bottom) were treated as indicated for 4 h and ROS levels were measured; N-acetyl cysteine (NAC, 10 mM) was used as a control to rescue ROS production in drug treated cells. Bars are the average of at least two independent experiments. Error bars represent SD. Statistical significance: n.s., not significant; ***p* < 0.01; ****p* < 0.001 (two-tailed unpaired Student’s *t*-test).

**A**

**B**

**C**

**Figure S9.** EGFR siRNA-mediated downregulation phenocopies cetuximab-mediated ROS production. (A), DiFi cells were transfected with anti-EGFR siRNA and EGFR expression was assessed after 48 and 72 hours by Western blotting. (B), Non-transfected cells where used for the control experiment with the anti-EGFR drug cetuximab**,** (**C**)**,** while EGFR-siRNA transfected cells were treated, the day after transfection, with ctrl media and vitamin C (VitC). The experiment was performed with technical duplicates for two independent biological replicates. One representative experiment is shown. Error bars represent SD. NT: Non-targeting siRNA. Treatments: ctrl, control media; VitC, 1 mM; Cetux, 50 μg/ml; combo, VitC 1 mM plus Cetux 50 μg/mL. Statistical significance: **p* < 0.05; (two-tailed unpaired Student’s *t*-test).

**A**

**B**

**Figure S10.** Vitamin C alone or in combination with cetuximab increases levels of lipid peroxidation in CRC cells. (**A**)**,** DiFi cells were treated with indicated agents for 24h and levels of malondialdehyde (MDA) were assessed to measure lipid peroxidation; (**B**) Ferrostatin (FRS-1) (2 μM) supplementation was used to rescue CRC cells from lipid peroxidation induced by combinatorial treatment. Bars represent the average of three independent experiments and error bars represent SD. Ctrl: control; VitC: vitamin C (1 mM); Cetux: cetuximab (50 µg/ml); combo: combination of VitC and cetuximab. Statistical significance: n.s., not significant; **p* < 0.05 (two-tailed unpaired Student’s *t*-test).

The whole western blot images of Figure 5C, Figure S4

**Figure 5C.** Raw Data.

**Figure S4.** Raw data.

| 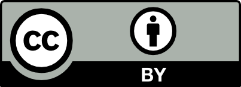 | © 2020 by the authors. Licensee MDPI, Basel, Switzerland. This article is an open access article distributed under the terms and conditions of the Creative Commons Attribution (CC BY) license (http://creativecommons.org/licenses/by/4.0/). |
| --- | --- |
